# Supplementary material for: Healing the Whole: An International Review of the Collaborative Care Model between Primary Care and Psychiatry
Source: Healthcare (Basel). 2024 Aug 22;12(16):1679. doi: 10.3390/healthcare12161679 (PMC11353489; doi:10.3390/healthcare12161679)
Supplement: Supplementary file 1 [file healthcare-12-01679-s001.zip › healthcare-3080035-supplementary.pdf]

Supplementary Material: Quality Assessment Report

Randomized Control Trials/Clinical Controlled Trials							
	**Bosanquet, K et al. [32]**	**Sadock, E et al. [30]**	**Aragonès, E et al. [22]**	**Schnurr, P.P. et al. [36]**	**Liao, S.-J. et al. [35]**	**Green, C. et al. [37]**	**Rossom, R.C. et al. [4]**
Do the study aim/purpose/objectives and inclusion/exclusion criteria assist in answering your clinical question?	Yes	Yes	Yes	Yes	Yes	Yes	Yes
Is a RCT or CCT congruent with the author's study aim, purpose, or objectives above?	Yes	Yes	Yes	Yes	Yes	Yes	Yes
Were the patients randomly assigned to treatment and control groups?	Yes	No	Yes	Yes	Yes	Yes	Yes
Was that randomization conducted appropriately?	Yes	N/A	Yes	Yes	Yes	Yes	Yes
At the start of the study, were the participants similar with respect to known factors of interest?	Yes	Yes	Yes	Yes	Yes	Yes	Yes
Aside from the experimental treatment, were the groups treated equally?	Yes	Yes	Yes	Yes	Yes	Yes	Yes
Were all patients who entered the study accounted for at its conclusion?	No	Yes	Yes	No	Yes	No	No
Were patients analyzed in the groups to which they were randomized?	Yes	Yes	Yes	Yes	Yes	Yes	Yes
Was the study process long enough to fully study effects of the intervention?	Yes	Yes	Yes	Yes	Yes	Yes	Yes
Were instruments used to measure the outcomes valid and reliable?	Yes	Yes	Yes	Yes	Yes	Yes	Yes
Was there freedom from conflict of interest?	Yes	Unknown	Yes	Yes	Yes	Yes	Yes
Did the study have a sufficiently large sample size?	Yes	Yes	Yes	Yes	Yes	Yes	Yes
Were the statistical analysis methods appropriate?	Yes	Yes	Yes	Yes	Yes	Yes	Yes
Were the results statistically significant?	Yes	Yes	Yes	No	Yes	Yes	No
Were results clinically significant?	Yes	Yes	Yes	Yes	Yes	Yes	Yes
Were adverse events assessed?	Yes	No	No	Yes	No	No	Yes
Can the results be applied to my population of interest?	Yes	Yes	Yes	Yes	Yes	Yes	Yes
Are my patient's and family's values and preferences satisfied by the treatment and its consequeces?	Yes	Yes	Yes	Yes	Yes	Yes	Yes
Would you include this study/article in development of a care recommendation?	Yes	Yes	Yes	Yes	Yes	Yes	Yes
Quality	Good quality RCT - 2a	Lesser Quality CCT [3b]	Good quality RCT - 2a	Good quality RCT - 2a	Good quality RCT - 2a	Good quality RCT - 2a	Good quality RCT - 2a

**Clinical Control Trials/ Cohort Studies**	
	**Truitt, F.E. et al. [31]**
Do the study aim/purpose/objectives and inclusion/exclusion criteria assist in answering your clinical question?	Yes
Is a CCT or cohort study congruent with the author’s study aim, purpose, or objectives above?	Yes
Were the data collected prospectively	No
Did the sample include an appropriate variety of patients to whom the index test will be applied to clinical practice?	Yes
Were the patients similar at the start of the trial, with respect to known prognostic factors?	Yes
Did patients receive the same reference standard, regardless of the index test?	Yes
Was the execution of the index test and the reference standard described?	Yes
Were all patients accounted for at the conclusion of the study?	No
Did the study have a sufficiently large sample size?	Yes
Was there freedom from conflict of interest?	Yes
Were the index test results and the reference standard results interpreted idepenedently?	No
Were the same clinical data available when test results were interpreted as would be available when the test is used in practice?	Yes
Were all test results reported, including uninterpretable or intermediate test results?	Yes
Can the results be applied to my population of interest?	Yes
Are my patient's and family's values and preferences satisfied by the treatment and its consequeces?	Yes
Would you include this study/article in development of a care recommendation?	Yes
Quality	Lesser Quality Cohort – Retrospective [4b]

Economic Analysis			
	**Green, C. et al. [37]**	**Angstman, K.B. et al. [29]**	**Camacho, E.M. et al. [28]**
Do the study aim/purpose/objectives and inclusion/exclusion criteria assist in answering your clinical question?	Yes	Yes	Yes
Is a decision analysis, economic analysis, or computer simulation congruent with the author's study aim, purpose, or objectives above?	Yes	Yes	Yes
Was a well-defined question posed?	Yes	Yes	Yes
Were all important, realistic strategies included and clearly specified?	Yes	Yes	Yes
Was there evidence that the intervention/strategy effectiveness had been established?	Yes	Yes	Yes
Were all important and relevant outcomes considered?	Yes	Yes	Yes
Was a model clearly described and appropriate?	Yes	Yes	Yes
For an economic analysis, do included costs match stated perspective(s)?	Yes	Yes	Yes
Were the outcomes and costs measured using valid and reliable tools?	Yes	Yes	Yes
In measuring outcomes and costs, were the measures/utilities used valued and appropriate?	Yes	Yes	Yes
Was an explicit and sensible process used to identify, select, and combine evidence into probabilities?	Yes	Yes	Yes
Was there freedom from conflict of interest?	Yes	Unkown	Yes
Does one strategy result in a clinically important gain for patients? If No, is the result a toss-up?	Yes	Yes	Yes
Could uncertainty in the evidence change the result?	No	No	No
For an economic analysis, was a comprehensive economic comparison of all important health care strategies conducted?	Yes	Yes	Yes
Was an incremental analysis of the outcomes and costs of alternatives performed?	Yes	Yes	Yes
Was appropriate allowance made for uncertainties in the analysis?	Yes	Yes	Yes
Were outcomes and costs adjusted for different times at which they occurred, such as discounting?	Yes	Yes	Yes
Are the estimates of outcomes and costs related to the baseline risk in the treatment population, if relevant?	Yes	Yes	Yes
Were the results statistically significant?	Yes	No	Yes
Were the results clinically significant?	Yes	Yes	Yes
Were the conclusions of the evaluation justified by the evidence presented?	Yes	Yes	Yes
Did the presentation and discussion of the results include all or enough of the issues that are of concern to consumers?	Yes	Yes	Yes
Can the results be applied to my population of interest?	Yes	Yes	Yes
Would you include this study/article in development of a recommendation?	Yes	Yes	Yes
Quality	Good Quality Economic Analysis [5a]	Good Quality Economic Analysis [5a]	Good Quality Economic Analysis [5a]

**Longitudinal Studies**						
	**Sederer, L.I. et al. [21]**	**Eghaneyan, B.H. et al. [33]**	**Standeven, L et al. [23]**	**Powers, D.M. et al. [34]**	**Bowen, D.J. et al. [38]**	**Moise, N. et al. [24]**
Do the study aim/purpose/objectives and inclusion/exclusion criteria assist in answering your clinical question?	Yes	Yes	Yes	Yes	Yes	Yes
Is a longitudinal study congruent with the author's study aim, purpose, or objectives above?	Yes	Yes	Yes	Yes	Yes	Yes
Were the study methods appropriate for the question?	Yes	Yes	Yes	Yes	Yes	Yes
Were instruments used to measure the outcomes valid and reliable?	Yes	Yes	Yes	Yes	Yes	Yes
Were all appropriate variables and interventions clearly described?	Yes	Yes	Yes	Yes	No	No
Were all appropriate outcomes clearly described?	Yes	Yes	Yes	Yes	No	Yes
Was there freedom from conflict of interest?	Unkown	Unkown	Yes	Yes	No	Yes
Were the statistical analysis methods appropriate?	Yes	Yes	Yes	Yes	Yes	Yes
Did the study have a sufficiently large sample size?	Yes	Yes	Yes	Yes	Yes	Yes
Were the results statistically significant?	Yes	Yes	Yes	Yes	Yes	Yes
Were the results clinically significant?	Yes	Yes	Yes	Yes	Yes	Yes
Were adverse events assessed?	No	Yes	No	No	No	No
Can the results be applied to my population of interest?	Yes	Yes	Yes	Yes	Yes	Yes
Are my patient's and family's values and preferences satisfied by the treatment and its consequeces?	Yes	Yes	Yes	Yes	Yes	Yes
Would you include this study/article in development of a care recommendation?	Yes	Yes	Yes	Yes	Yes	Yes
Quality	Lesser Quality Longitudinal Study [4b]	Good Quality Longitudinal Study [4a]	Good Quality Longitudinal Study [4a]	Good Quality Longitudinal Study [4a]	Lesser Quality Longitudinal Study [4b]	Good Quality Longitudinal Study [4a]

**Qualitative Studies**				
	**Bosanquet, K et al. [32]**	**Sederer, L.I. et al. [21]**	**Powers, D.M. et al. [34]**	**Moise, N. et al. [24]**
Do the study aim/purpose/objectives and inclusion/exclusion criteria assist in answering your clinical question?	Yes	Yes	Yes	Yes
Is a qualitative study congruent with the author's study purpose above?	Yes	Yes	Yes	Yes
Was the qualatiative design clearly identified?	Yes	Yes	Yes	Yes
Was the area of study clearly stated in one sentence?	Yes	No	Yes	Yes
Was the design appropriate to explore the area of study being studied?	Yes	Yes	Yes	Yes
Was a guiding framework identified?	Yes	No	No	Yes
Was the guiding framework appropriate for the area of study being evaluated?	Yes	No	No	Yes
Was the setting clearly identified for the area of study being studied?	Yes	Yes	Yes	Yes
Was the context of the participants analyzed using the words of the participants?	Yes	Yes	Yes	Yes
Was the researcher known and trusted by the participants?	Yes	Unkown	Unkown	Unkown
Did the researchers report how findings were confirmed?	Yes	Yes	Yes	Yes
Did the researchers discuss the essence of the findings within the socio-cultural context?	Yes	Yes	Yes	Yes
Was saturation of data discussed and reached?	No	No	No	Yes
Was the data analysis method identified?	Yes	No	No	Yes
Were the themes reported in terms of the theoretical framework?	Yes	N/A	N/A	Yes
Was this information gained from the study applicable to similar groups and contexts?	Yes	Yes	Yes	Yes
Quality	Good Quality Qualitative Study [2a]	Lesser Quality Qualitative Study [2b]	Lesser Quality Qualitative Study [2b]	Good Quality Qualitative Study [2a]

**Mixed Method Studies**					
	**Bosanquet, K et al. [32]**	**Sederer, L.I. et al. [21]**	**Powers, D.M. et al. [34]**	**Green, C. et al. [37]**	**Moise, N. et al. [24]**
Were two different methods or approaches used in the study?	Yes	Yes	Yes	Yes	Yes
If applicable, was (were) the qualitative components of the study welldeveloped, based on appraisal using the Meaning/KAB – Qualitative Study Evidence Appraisal Form?	Yes	No	No	N/A	Yes
If applicable, was (were) the quantitative components of the study welldeveloped, based on appraisal using the appropriate Evidence Appraisal Form for that study design?	Yes	No	Yes	Yes	Yes
Were the two components used to inform each other for joined, comprehensive results or discussion?	Yes	Yes	Yes	Yes	Yes
Were the mixed results significant?	Yes	Yes	Yes	Yes	Yes
Can the results be applied to my population of interest?	Yes	Yes	Yes	Yes	Yes
Are my patient's and family's values and preferences satisfied by the knowledge gained from this study?	Yes	Yes	Yes	Yes	Yes
Would you include this study/article in development of a reccomendation?	Yes	Yes	Yes	Yes	Yes
Quality	Good Quality Mixed Methods Study - 2a/2a	Lesser Quality Mixed Methods Study 2b/4b	Good Quality Mixed Methods Study - 2a/4b	Good Quality Mixed Methods Study 2a/5a	Good Quality Mixed Methods Study 2a/4a
